# Machine Learning-Based Classification of Human Behaviors and Falls in Restroom via Dual Doppler Radar Measurements †

**DOI:** 10.3390/s22051721

**Published:** 2022-02-22

**Authors:** Kenshi Saho, Sora Hayashi, Mutsuki Tsuyama, Lin Meng, Masao Masugi

**Affiliations:** 1 Department of Intelligent Robotics, Toyama Prefectural University, Imizu 939-0398, Japan; 2 Department of Electronic and Computer Engineering, Ritsumeikan University, Kusatsu 525-8577, Japan; sora8840817@gmail.com (S.H.); ri0082ip@ed.ritsumei.ac.jp (M.T.); menglin@fc.ritsumei.ac.jp (L.M.); masugi@fc.ritsumei.ac.jp (M.M.)

**Keywords:** Doppler radar application, restroom, fall detection, human behavior classification, remote monitoring

## Abstract

This study presents a radar-based remote measurement system for classification of human behaviors and falls in restrooms without privacy invasion. Our system uses a dual Doppler radar mounted onto a restroom ceiling and wall. Machine learning methods, including the convolutional neural network (CNN), long short-term memory, support vector machine, and random forest methods, are applied to the Doppler radar data to verify the model’s efficiency and features. Experimental results from 21 participants demonstrated the accurate classification of eight realistic behaviors, including falling. Using the Doppler spectrograms (time–velocity distribution) as the inputs, CNN showed the best results with an overall classification accuracy of 95.6% and 100% fall classification accuracy. We confirmed that these accuracies were better than those achieved by conventional restroom monitoring techniques using thermal sensors and radars. Furthermore, the comparison results of various machine learning methods and cases using each radar’s data show that the higher-order derivative parameters of acceleration and jerk, and the motion information in the horizontal direction are the efficient features for behavior classification in a restroom. These findings indicate that daily restroom monitoring using the proposed radar system accurately recognizes human behaviors and allows early detection of fall accidents.

## 1. Introduction

Aging is a global phenomenon responsible for various problems related to shortened health expectancy and the sudden death of elderly people [1]. Therefore, monitoring systems for early detection of accidents and abnormal behaviors in elderly adults, such as falling, have recently been developed based on sensors and Internet-of-Things technologies [2]. However, such systems are not used inside restrooms due to privacy concerns. The early detection of falls and abnormal behaviors in restrooms is important because it is one of the dangerous spaces in the home where elderly people are likely to fall [3]. Even though accelerometry-based approaches for fall detection in restrooms have been proposed [4,5], they require the subjects to wear the sensor devices.

Few studies have investigated camera-based approaches for the remote monitoring of restrooms [6] based on the image-processing-based fall detection technique [7,8]. However, their measurement accuracy depends on lighting conditions and the subjects’ clothing. In addition, installing cameras in restrooms is challenging because of privacy issues. Hence, investigations of restroom monitoring have been limited. Even though infrared, thermal, sensor-based posture estimation in restrooms has been proposed [9,10], its measurement accuracy depends on the temperature of the toilet and of the subject’s clothing.

Radar technology is a promising candidate method for solving the above-described problems because it does not invade privacy during measurement and is not affected by temperature conditions or clothing. Therefore, radar-based motion recognition and fall detection are active research areas. Various approaches based on machine learning of radar images, such as time–Doppler, time–range, or range–Doppler images, have been proposed and demonstrated in realistic situations [11,12,13]. However, research on the use of radar in restrooms is quite limited. Though the detection of falls in restrooms using radar systems or the classification of normal and abnormal behaviors by range (distance) information [14,15] have been studied based on classical signal detection or discriminant analysis methods, it is difficult to determine what is happening in a restroom because only abnormal behaviors, such as falls, are detected. Moreover, their accuracy and practicality were both insufficient. To solve these problems, our recent paper reported the long short-term memory (LSTM)-based classification of eight types of behaviors and falls in a restroom with approximately 80% accuracy using the Doppler radar-measured velocity time series [16].

As a significant extension of our previous study [16], this study presents a more accurate classification using the convolutional neural network (CNN)-based approach and investigates the accurate classification of human behavior in restrooms via various machine-learning methods. The contributions of this study are as follows:

- The efficient implementation of Doppler radars and experimental examples for privacy-protected restroom monitoring were provided for the realistic environment; this is a significant contribution because there are only several limited reports on realistic restroom monitoring.
- Efficient classification models and types of their input data for the radar-based restroom-monitoring system were clarified via the thorough comparison of the radar-based motion recognition approaches.
- The classification of human behaviors and falls in a restroom with above 95% was demonstrated. This result shows significant improvement over other conventional studies on radar-based restroom monitoring [14,15,16].

This paper is an extended version of our conference paper [17] that simply presented the results for the CNN-based approach. In this study, we added the results for the comparison with other various machine learning-based classification methods, the details of the implementation of the classification methods, comparison with other conventional studies, and investigation to elucidate the efficient features.

## 2. Related Work

The machine learning-based human motion classification has been widely investigated in various sensing technologies such as cameras, depth sensors, and accelerometers [18,19,20,21]. For various applications, the practicality and versatility of various machine-learning models [22,23,24] have been demonstrated.

For the field of radar technology, the machine learning methods that have been established for the abovementioned studies have also been applied to human motion recognition using the Doppler and/or range information obtained via radar sensing [11,25,26]. For example, radar-based fall detection has been widely studied [27,28] and various efficient methods using machine learning techniques have been proposed [29,30]. In recent years, accurate fall detections using radars have been achieved with machine learning methods such as CNN [12,31,32,33,34], LSTM [13,34], random forest (RF) [35], and support vector machine (SVM) [36]. These classification techniques are properly selected based on the features of the problem and the objectives of the radar data analysis. The CNN-based classification technique has achieved the accurate classification of human motion using Doppler radar spectrograms [11,12,35,37]. Additionally, the LSTM-based technique has achieved better accuracy for the continuous classification problem [13,37,38]. Although these techniques can achieve relatively accurate classifications, the mechanisms and/or factors of the classifications are generally unclear. Thus, the classical motion parameter-based approaches are still important in contemporary radar technology for developing the motion recognition system whose mechanism and performance are guaranteed [29,37,39,40]. The methodology for the design of the radar-based motion recognition system using these various machine learning approaches is being established for various types of experimental data.

However, as stated in the Introduction, most studies on motion recognition and fall detection studies using radar have not considered their application to the restroom. Thus, there are only several studies on radar-based restroom monitoring [14,15,16]. For example, in [15], the classification accuracy of seven types of behaviors and falls was approximately 60%. The low accuracy of conventional radar systems may be due either to the use of range information or to the use of classical detection/classification methods. Thus, the efficient machine learning methods that are suitable for radar-based restroom monitoring and their appropriate input data have not been investigated at all. For this purpose, our previous study [16] achieved accurate classification of eight human behaviors and falls in the restroom. This study aimed to extend this previous study with respect to the classification accuracy and to clarify the efficient models and features for behavior classification in radar-based restroom monitoring.

## 3. Experiments for Dataset Generation

### 3.1. Doppler Radar Experiments

**Figure 1:**
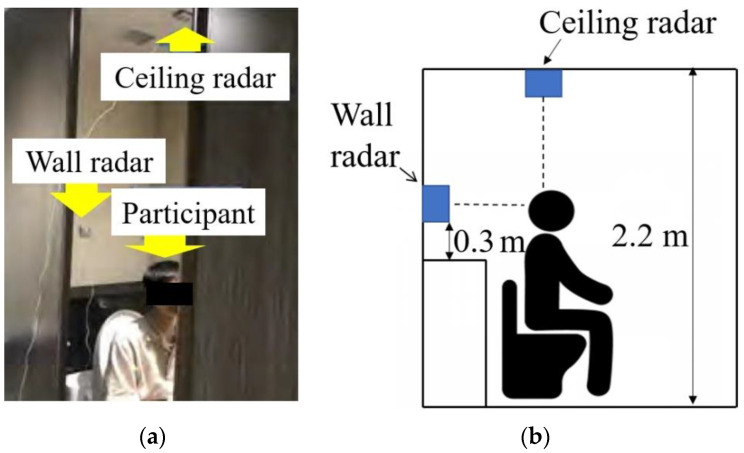
Doppler radar sensing system for measuring behaviors in a restroom. (**a**) Experimental site and (**b**) measurement setup.

Figure 1 shows the experimental site and outline of the measurement system. Twenty-one healthy young men (age: 22.4 ± 1.1 years, height: 173.8 ± 5.1 cm) consented to participate in this study and were instructed regarding the testing procedures prior to the experiments. Informed consent was obtained from all participants. Each participant performed the following eight types of behaviors three times: (a) opening the toilet lid, (b) pulling down the pants, (c) sitting, (d) taking the toilet paper, (e) standing, (f) pulling up the pants, (g) closing the toilet lid, and (h) falling. Falling is defined as the motion of falling forward from a seated position, which is one of the realistic falling motions in restrooms [6]. For example, a person seated on the toilet falls when leaning forward slowly.

We used 24 GHz continuous-wave radars (ILT office, BSS-110) with ±14° plane directivity mounted as shown in Figure 1. The radars were installed above (ceiling radar) and behind (wall radar) the participant. The ceiling and wall radars measured the motion along the vertical and horizontal directions, respectively. The Doppler radar transmitted a 24 GHz sinusoidal wave with an effective isotropic radiated power of 40 mW to each participant. The demodulated in-phase/quadrature radar signal *s*(*t*) was obtained using a quadrature detector and an analog-to-digital converter with a sampling frequency of 600 Hz, and a measurement velocity range of −1.875–1.875 m/s.

### 3.2. Generation of Spectrogram Dataset

Similar to our previous study [41], the short-time Fourier transforms (STFT) of the received signals were calculated to generate the spectrogram images (time–velocity–power distribution images) as follows. First, we removed the zero-Doppler frequency components from the received signals using a one-dimensional Butterworth high pass filter with a cutoff frequency of 30 Hz to eliminate echoes from static objects such as walls and toilet seats. The STFT of *s*(*t*) was calculated as *S*(*t*, *f*_d_) = ∫s(τ)*w*(τ − *t*)exp(−j2π*f*_d_*τ*)d*τ*, where *t* is the time, *f*_d_ is the Doppler frequency, and *w*(*t*) is the window function. For *w*(*t*), the Hamming window function, with a length of 213.3 ms (corresponding to the frequency resolution Δ*f*_d_ = 1/(213.3 × 10^−^^3^) = 4.69 Hz) and overlap length of 211.6 ms, was empirically used for the STFT process. Doppler velocity *v*_d_ was calculated with *v*_d_ = *cf*_d_/(2*f*_0_), where *f*_0_ is the frequency of the transmitting signal (24.0 GHz) and c is the speed of light (2.999 × 10^8^ m/s). Using this equation, we obtained the spectrogram |*S*(*t*, *v*_d_)|^2^ (based on the above setting, the resolution of *v*_d_ in the spectrogram was Δ*v*_d_ = *c*Δ*f*_d_/(2*f*_0_) = 0.0293 m/s). Finally, we removed the components with a received power density of less than 0 dB/Hz, assuming that these components corresponded to random noises.

**Figure 2:**
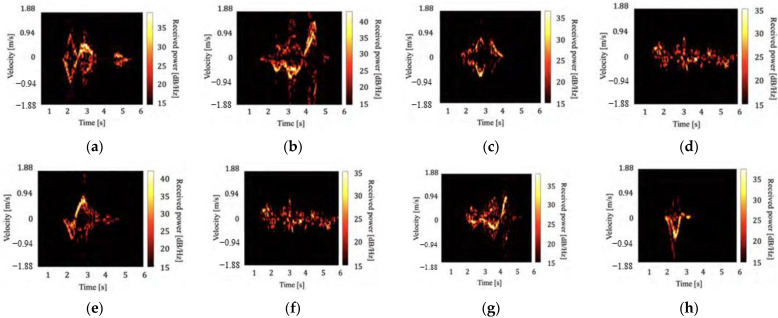
Examples of spectrograms measured with the ceiling radar. (**a**) Opening the toilet lid, (**b**) pulling down the pants, (**c**) sitting, (**d**) taking the toilet paper, (**e**) standing, (**f**) pulling up the pants, (**g**) closing the toilet lid, and (**h**) falling.

**Figure 3:**
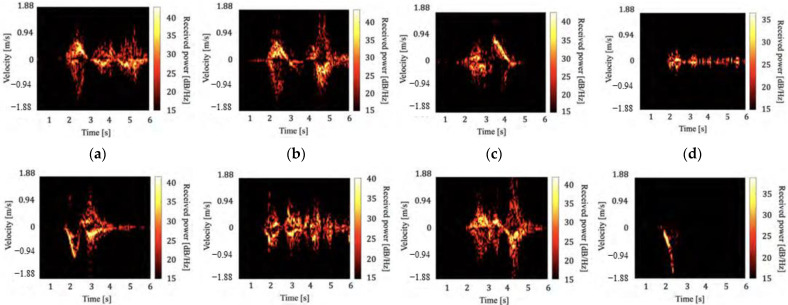
Examples of spectrograms measured with the wall radar. (**a**) Opening the toilet lid, (**b**) pulling down the pants, (**c**) sitting, (**d**) taking the toilet paper, (**e**) standing, (**f**) pulling up the pants, (**g**) closing the toilet lid, and (**h**) falling.

Figure 2 and Figure 3 show examples of the generated spectrograms for all behaviors of the ceiling and wall radars, respectively. The motion toward the ceiling and back of the subject is positive for the ceiling and wall radars that measure the head and torso, respectively. For example, we confirmed the significant negative velocity components in Figure 2f and Figure 3f that correspond to the fall forward motion for behavior (f). Similarly, the motion characteristics of each of the other behaviors could also be confirmed. For behaviors (d) and (f), no characteristic velocity components were obtained because they did not involve large motions compared with the other behaviors. Although the different features of each behavioral spectrogram were confirmed to some extent, some behaviors were difficult to classify. For example, the spectrograms of the behaviors (b) and (e) in Figure 2 have relatively similar characteristics. Therefore, this study aimed to classify the behaviors using various machine learning methods.

## 4. Implementation of the Machine Learning-Based Classification Methods

This study implemented three types of classification methods and compared their accuracy to determine the most efficient method and investigate the features for efficiently classifying behaviors and falls in a restroom.

### 4.1. Spectrogram Image-Based Method Using CNN

**Figure 4:**
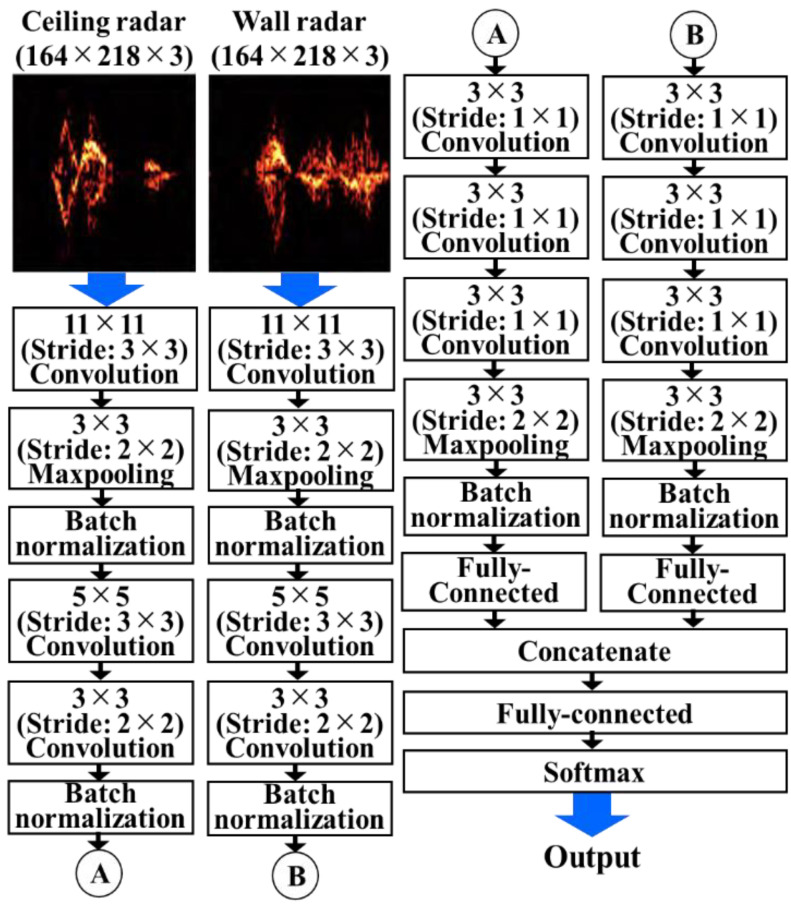
Process and structure of the CNN method.

This method (CNN method) directly uses the generated spectrogram images as the CNN input. Figure 4 shows the process and structure of the network. The spectrogram PNG images of size 168 × 218 generated from the two radars were input into the CNN. CNN has a structure similar to AlexNet [22]. However, to avoid overfitting, we used the batch normalization layer instead of the dropout layer. To fuse the two images obtained from the dual radars, we constructed two similar CNNs and combined their outputs using the concatenate layer that was then used in the fully-connected layer to determine the output class. A stochastic gradient descent with a momentum optimization algorithm was used for the network optimization. We trained for 100 epochs with a learning rate of 0.01 and batch size of 8. In addition, to compare the classification accuracy with the single and dual radars, the input from a single spectrogram image was fed into the CNN structure without the concatenate layer.

This classification method using spectrogram images and CNN is known as the most efficient method for various applications of radar motion classification [11]. Although the efficient input data and CNN structure depend on the applications, many studies demonstrated the best accuracy with the CNN-based method compared to the use of other classifiers. However, existing studies fail to explain the classification mechanism or to determine the efficient features. Therefore, this study compared the proposed CNN method with other methods to determine the efficient features and reasons for classification.

### 4.2. Spectrogram Envelope-Based Method Using LSTM

**Figure 5:**
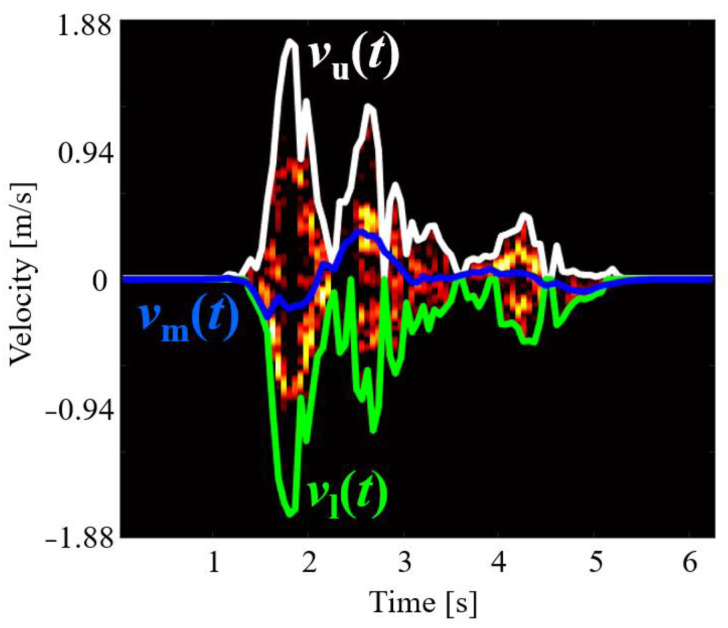
Example of extraction of spectrogram envelopes.

**Figure 6:**
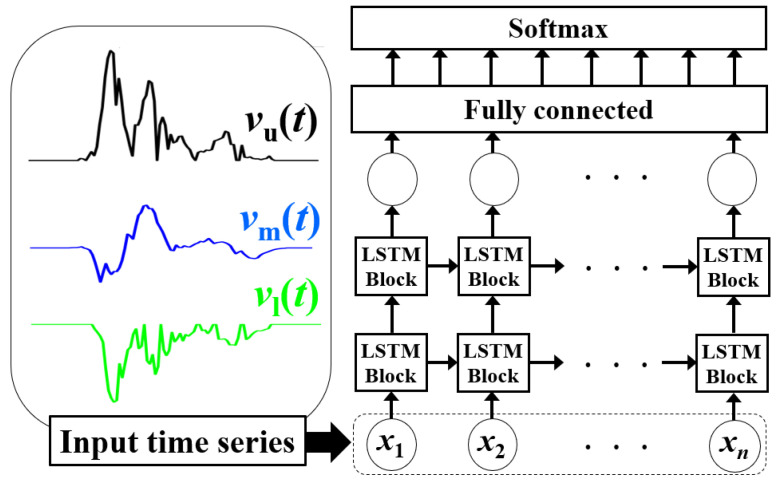
Process and structure of the LSTM method.

The LSTM method uses the velocity time-series spectrogram envelopes, as shown in Figure 5, for classification [16]. We extracted three types of envelopes from the spectrograms with the same process as in [41]: the upper envelope *v*_u_(*t*), lower envelope *v*_l_(*t*), and power-weighted mean velocity *v*_m_(*t*). These extracted envelopes were then input into the LSTM as outlined in Figure 6. The data length of each envelope was 102 points, and the dimensions of the input data for the single and dual radar fusion were 102 × 3 and 102 × 6, respectively. We empirically optimized the hyperparameters using an Adam optimizer [23]. Thus, the number of hidden layers was 100, batch size was 64, learning rate was 0.001, and number of epochs for the training was 300.

### 4.3. Motion Parameter-Based Methods

**Figure 7:**
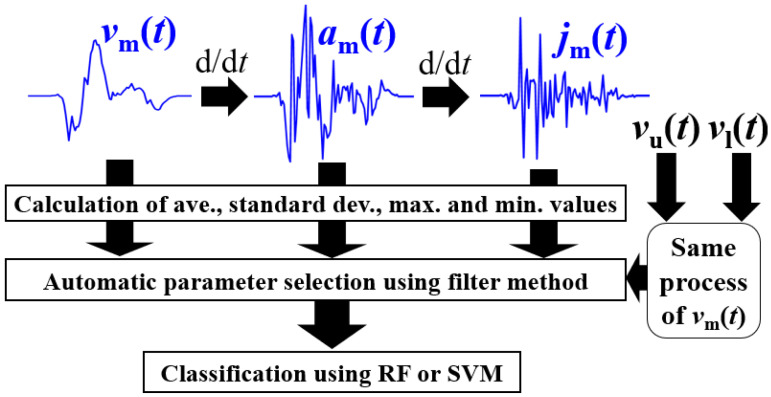
Outline of the RF and SVM methods using the motion parameters.

These methods use kinematic parameters extracted using the spectrogram envelopes for classification and can directly obtain efficient motion-feature parameters for behavior classification, as shown in Figure 7. First, we extracted the three envelopes *v*_u_(*t*), *v*_l_(*t*), and *v*_m_(*t*), as with as the LSTM method. We calculated four representative values of mean, maximum, minimum, and standard deviation with respect to time for each envelope. Then, we calculated the time derivative of the envelopes to obtain the acceleration and jerk time series. For example, for *v*_m_(*t*), we calculated the acceleration time series *a*_m_(*t*) = d*v*_m_(*t*)/d*t* and jerk time series *j*_m_(*t*) = d*a*_m_(*t*)/d*t*. Empirically designed, moving-average low pass filters with an average length of 0.15 s were used to remove small errors in each time series. Similar to the velocity time series, we also calculated the four representative values of *a*_m_(*t*) and *j*_m_(*t*). The same process was also applied to *v*_u_(*t*) and *v*_l_(*t*). Thus, we extracted 4 (parameters) × 3 (envelopes) × 3 (time series of velocity, acceleration, and jerk) = 36 parameters for each radar. For dual radar fusion, 36 × 2 = 72 parameters were obtained as candidate feature parameters for classification. From these parameters, efficient feature parameters for each classifier were automatically selected using the filter method [24]. The filter method determines the relevance of each parameter for classification, and the top 20% is selected. We selected the widely used random forest (RF) and support vector machine (SVM) as the classifiers in this study. Their hyperparameters were optimized using a grid search, and the Gaussian kernel was used for the SVM.

## 5. Evaluation and Discussion

### 5.1. Main Evaluation Results

We evaluated and compared the classification accuracy of the four classification methods in Section III using hold-out validation. For all of the methods, we also compared the accuracy for three cases: only ceiling radar data, only wall radar data, and fused data from the two radars. The classification model was first trained using 80% of the data for each case and then tested with the remaining 20%. We performed 30 trials of the test processes by randomly varying the training data.

Table 1 summarizes the mean and standard deviation of classification accuracies from the 30 test trials for the four classification methods. The CNN method achieved the best accuracy of 95.6%, indicating that the spectrogram images were more effective than the spectrogram envelope [16] or motion parameter-based approach for human behavior and fall classification in restrooms. However, other classification methods also achieved moderate accuracy, making it possible to obtain efficient motion information and/or parameters necessary for classification. This is discussed in the next subsection.

Furthermore, better accuracy was obtained when using dual radar data than by using the ceiling or wall radar data only. In particular, a significant improvement was obtained for the CNN and RF methods when dual radar data were used. Therefore, we conclude that the motions in both upward and horizontal directions included the differences in the assumed behaviors and falls.

**Figure 8:**
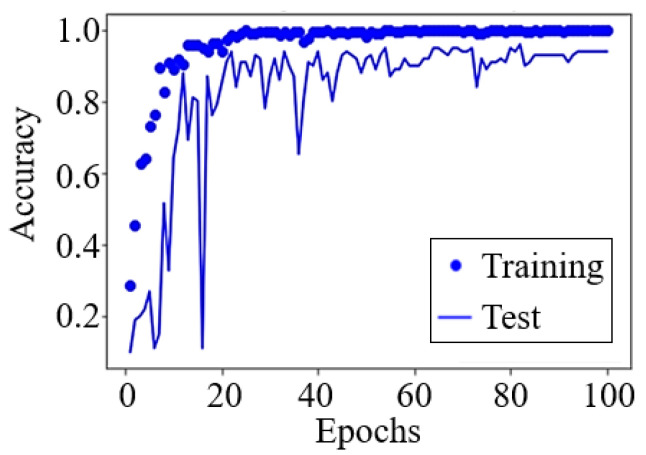
Sample learning curve of the CNN method using the dual radar data.

The results from the CNN method based on the convergence curve are shown in Figure 8, and the confusion matrix is further discussed to validate its performance. No overfitting was observed in either test or training processes. The accuracy in the test process converged in less than 50 epochs. Table 2 shows the confusion matrices for the data from the ceiling, wall, and dual radars. The classification accuracies of “(f) pulling up the pants” and “(b) pulling down the pants” are worse for the ceiling and wall radar data, respectively. However, the classification accuracy of (f) improves when the fused data are considered whereas that of (b) is not improved. The classification accuracy of “(h) falling” is 100% in all cases, and is the most important function for the practical use of fall detection.

### 5.2. Discussion on Efficient Features

This section discusses the efficient features measured with each radar when classifying human behaviors in restrooms. First, we discuss the effectiveness of the data from each radar and the fused data. Table 3, Table 4 and Table 5 show the confusion matrices for the LSTM, RF, and SVM methods, respectively. As indicated in the confusion matrices of the CNN (Table 2) and LSTM methods, all behaviors and falls are accurately classified using deep learning methods. However, we can see the different classification accuracies for some classes. For example, as indicated in Table 2, the classification accuracy of the classification of behaviors (b) and (g) was worse in the results of the CNN method with the ceiling radar. In contrast, these were accurately classified with the LSTM method with the ceiling radar data as shown in Table 3. These results indicate that some of the behaviors accurately classified by these methods varied because of the differences in the included features in the spectrogram images and envelopes. In addition, for the motion parameter-based methods (the RF and SVM methods), behaviors (b) and (g) were classified with better accuracy, as indicated in Table 4 and Table 5, even though the overall accuracies were significantly worse than the CNN method. Because the motion parameters were extracted from the envelopes that were also used in the LSTM method, the efficient features for the classification might be included in the spectrogram envelopes extracted from dual radars. In the following, we discuss the efficient features and factors of our results.

Next, we discuss the effectiveness of using dual radar data. Similar to the results for the CNN method, better performance was observed with the wall radar data than with the ceiling radar data. These results indicate that the motion information in the horizontal direction obtained with the wall radar includes significant information for classifying the assumed human behaviors. Another reason is that the wall radar received the data for the whole body, whereas the ceiling radar mainly obtained the motion of the head. The confusion matrices further confirm the differences between the two radars’ results for the classified behaviors. In particular, the confusion matrices of the RF and SVM methods in Table 4 and Table 5 indicate that the combination of the data from the two radars significantly improves the classification accuracy because the data from the two radars complement each other. Similar accuracy improvements based on the fusion of dual radar data also can be confirmed from the confusion matrices of other methods, further verifying the effectiveness of the dual radar data.

We now discuss the efficient features included in the spectrograms. Because the classification accuracy of the eight behaviors with RF and SVM methods was above 60%, we consider the selected feature parameters for these methods. Table 6 shows the selected features for the RF and SVM methods using the filter method. The acceleration and jerk parameters were selected for all radar cases. These results indicate that the detailed motion parameters of acceleration and jerk were more effective than the velocity parameters obtained directly from Doppler radar measurements. However, the LSTM and CNN methods performed better than the RF and SVM methods using the motion parameters.

We conclude that deep learning can grasp the detailed information in the spectrograms corresponding to higher-order derivative parameters. In addition, because the CNN method had better accuracy than the LSTM method, detailed motion information was obtained from the main components extracted as the spectrogram envelopes and from other components corresponding to the micromotion of the various body parts.

The findings regarding the efficient features for the classification of human behaviors and falls in restrooms are summarized as follows:

- The wall radar that measured motion in the horizontal direction was more effective than the ceiling radar that measured motion in the vertical direction.
- The accurately classified classes for the two radars were different. Hence, a fusion of the two radars was effective.
- The proposed method effectively used the detailed higher-order derivative parameters of acceleration and jerk.
- Detailed motion information was diffused across the whole of the spectrograms and was not limited to the main components, and was efficiently extracted via the CNN.

However, as the limitation of this study, the concrete clarification of the efficient parameters and/or factors for our classification problem was difficult. To achieve this, we have to find the features that indicate clear divergence of the assumed behaviors in restrooms based on other various approaches (e.g., using principal component analysis, application of other classification algorithms and comprehensive comparison with the results of this study, and data acquisition from a larger number of participants).

### 5.3. Comparison with Conventional Studies

In this section, we compare our method with the conventional remote sensor-based monitoring methods for restrooms. Table 7 outlines the comparison of the experimental studies aimed at detecting abnormal, dangerous behaviors in restrooms. The proposed method achieved the best performance in terms of the classification accuracy, number of classified behaviors, number of participants, and detection accuracy of the human fall.

Due to privacy issues, the number of studies on restroom monitoring using cameras is quite limited. Reference [6] is one of the few studies that report on camera-based monitoring of restrooms to detect dangerous situations to protect the elderly. However, because sensors without privacy issues are more suitable for restroom monitoring, approaches using infrared-based thermal sensors and radars have been recently studied. However, most studies classify situations as normal or dangerous behaviors [9,10]. Although thermal sensors show a sufficiently accurate classification, detailed behaviors were not classified because the sensors cannot detect the motion information directly. By contrast, radar techniques can acquire motion velocity information and classify it into multiple behaviors as carried out in [14,15]. However, the accuracy achieved in [15] was insufficient because only the simple feature parameters related to distance and signal information and the RF method were used. Therefore, our previous study [16] proposed the LSTM method that used the rich velocity information obtained via spectrogram envelopes. While both our previous research and the present study can classify the behaviors into eight categories, the proposed method was carried out using CNN and showed higher classification accuracy, including 100% fall detection. In addition, the present study used a relatively large dataset generated from a larger number of participants, and the spectrogram images utilized the rich velocity information included in the Doppler radar signals.

## 6. Conclusions

This study used Doppler radar technology to classify the behaviors and falls in a restroom based on machine learning approaches. The CNN, LSTM, SVM, and RF methods were applied and compared to determine the most efficient method and features for restroom monitoring. Furthermore, dual radars mounted on the ceiling and wall of a restroom collected motion information in horizontal and vertical directions. The experimental results revealed that the CNN method using the spectrogram images as input achieved the best accuracy of 95.6% when classifying the eight behaviors of 21 participants. In addition, the classification rate of the fall and other behaviors was 100%. These results indicate that the proposed Doppler radar system can accurately recognize behaviors and detect falls in a restroom without any privacy concerns. In addition, we identified efficient features in the motions by comparing the four machine learning methods using single and dual radar data. The motion information corresponding to the higher-order derivative parameters of acceleration and jerk in the horizontal direction was efficient, and the corresponding features were extracted via the CNN method.

However, this study had the following limitations. The motion features of the efficient classification were insufficiently revealed in this study, as discussed in Section 5.2. In addition, only young people participated and eight limited behaviors were assumed.

Thus, further experiments are needed to address the above limitations of this study. In the future, more studies are needed with elderly participants. Other behaviors and falls, such as using a smartphone or falling sideways, should be considered. In addition, combining multiple models, such as LSTM and CNN, may improve classification accuracy because some of the behaviors accurately classified by the LSTM and CNN methods varied. The model combination also may lead to the clarification of the efficient features for behavior classification in restrooms. Furthermore, the use of multiple radars (more than two) is an important future study area.

## Figures and Tables

**Table 1:** Summary of Classification Results.

Method	Ceiling Radar Data	Wall Radar Data	Both Radars
RF	41.5 ± 4.82%	55.2 ± 3.80%	63.8 ± 3.72%
SVM	60.4 ± 5.37%	62.4 ± 4.31%	63.4 ± 4.27%
LSTM [16]	72.3 ± 4.96%	82.6 ± 4.24%	83.2 ± 3.93%
CNN	90.3 ± 2.66%	91.5 ± 3.07%	95.6 ± 2.28%

**Table 2:** Confusion matrix of the CNN method.

		Predicted Label							
		(a)	(b)	(c)	(d)	(e)	(f)	(g)	(h)
True Label	(a)	0.90/	0/	0/	0/	0/	0/	0.10/	0/
		0.93/	0/	0/	0/	0/	0/	0.07/	0/
		1	0	0	0	0	0	0	0
	(b)	0/	0.85/	0/	0.15/	0/	0/	0/	0/
		0/	0.77/	0/	0/	0/	0/	0.23/	0/
		0	0.79	0	0	0	0	0.21	0
	(c)	0.08/	0/	0.92/	0/	0/	0/	0/	0/
		0/	0/	1/	0/	0/	0/	0/	0/
		0	0	1	0	0	0	0	0
	(d)	0/	0/	0/	1/	0/	0/	0/	0/
		0/	0/	0/	0.9/	0.1/	0/	0/	0/
		0	0	0	1	0	0	0	0
	(e)	0/	0/	0/	0/	0.92/	0.08/	0/	0/
		0/	0/	0/	0/	1/	0/	0/	0/
		0	0	0	0	1	0	0	0
	(f)	0/	0/	0/	0.22/	0.06/	0.72/	0/	0/
		0/	0/	0/	0/	0/	0.92/	0.08/	0/
		0.07	0	0.07	0	0	0.86	0	0
	(g)	0.08/	0/	0/	0/	0/	0/	0.92/	0/
		0/	0/	0/	0/	0/	0/	1/	0/
		0.08	0	0	0	0	0	0.92	0
	(h)	0/	0/	0/	0/	0/	0/	0/	1/
		0/	0/	0/	0/	0/	0/	0/	1/
		0	0	0	0	0	0	0	1

Each cell represents the results for ceiling/wall/dual radars.

**Table 3:** Confusion matrix of the LSTM method.

		Predicted Label							
		(a)	(b)	(c)	(d)	(e)	(f)	(g)	(h)
True Label	(a)	0.71/	0/	0/	0/	0/	0.29/	0/	0/
		0.90/	0/	0/	0/	0/	0/	0.10/	0/
		0.62	0	0	0	0	0.31	0.077	0
	(b)	0.091/	0.27/	0.091/	0.18/	0/	0/	0.36/	0/
		0/	0.6/	0.2/	0/	0.067/	0.067/	0/	0.067/
		0	0.83	0.083	0.083	0	0	0	0
	(c)	0/	0.083/	0.83/	0/	0/	0/	0/	0.083/
		0.11/	0/	0.89/	0/	0/	0/	0/	0/
		0.07	0	0.93	0	0	0	0	0
	(d)	0/	0.071/	0/	0.86/	0/	0/	0.071/	0/
		0.083/	0/	0/	0.92/	0/	0/	0/	0/
		0	0	0	1	0	0	0	0
	(e)	0/	0/	0.11/	0/	0.78/	0.056/	0/	0.056/
		0/	0/	0/	0/	0.94/	0.06/	0/	0/
		0	0	0	0	0.78	0.11	0.11	0
	(f)	0.25/	0.13/	0/	0/	0/	0.38/	0.25/	0/
		0.11/	0/	0.11/	0/	0/	0.78/	0/	0/
		0.08	0	0.07	0	0.17	0.75	0	0
	(g)	0/	0.16/	0/	0.077/	0/	0.077/	0.62/	0.077/
		0/	0.17/	0/	0/	0/	0.083/	0.75/	0/
		0	0.14	0.07	0	0	0	0.79	0
	(h)	0.059/	0/	0/	0/	0/	0/	0/	0.94/
		0/	0/	0/	0/	0/	0/	0/	1/
		0	0	0.08	0	0	0	0	0.92

Each cell represents the results for ceiling/wall/dual radars.

**Table 4:** Confusion matrix of the RF method.

		Predicted Label							
		(a)	(b)	(c)	(d)	(e)	(f)	(g)	(h)
True Label	(a)	0.64/	0.091/	0/	0.18/	0.091/	0/	0/	0/
		0.45/	0.27/	0.091/	0/	0/	0.11/	0/	0/
		0.62	0.23	0	0	0	0.15	0	0
	(b)	0/	0.21/	0.14/	0.21/	0.21/	0.071/	0.071/	0.071/
		0/	0.78/	0.11/	0/	0/	0.11/	0/	0/
		0	0.71	0	0.14	0	0.071	0	0.071
	(c)	0/	0/	0.45/	0/	0.45/	0/	0/	0.091/
		0.059/	0.059/	0.59/	0.18/	0.059/	0.059/	0/	0/
		0.1	0	0.65	0	0.25	0	0	0
	(d)	0.083/	0/	0/	0.5/	0.083/	0.17/	0.083/	0.083/
		0/	0/	0/	0.78/	0.22/	0/	0/	0/
		0	0	0.2	0.6	0.067	0.067	0.067	0
	(e)	0/	0/	0.1/	0.1/	0.6/	0.1/	0.1/	0/
		0/	0/	0.091/	0.45/	0.27/	0/	0.091/	0.091/
		0	0	0	0	1	0	0	0
	(f)	0.31/	0/	0/	0.15/	0.077/	0.23/	0.15/	0.077/
		0.043/	0.26/	0/	0.043/	0.26/	0.35/	0.043/	0/
		0.091	0	0.091	0.091	0.18	0.27	0.27	0
	(g)	0.15/	0.15/	0.15/	0/	0.15/	0/	0.15/	0.077/
		0/	0.36/	0.21/	0.071/	0.071/	0/	0.29/	0/
		0	0.29	0.071	0	0.071	0.071	0.43	0.071
	(h)	0.24/	0/	0.29/	0.059/	0.12/	0.059/	0/	0.24/
		0/	0/	0/	0/	0/	0/	0/	1/
		0	0	0	0	0	0	0	1

Each cell represents the results for ceiling/wall/dual radars.

**Table 5:** Confusion matrix of the SVM method.

		Predicted Label							
		(a)	(b)	(c)	(d)	(e)	(f)	(g)	(h)
True Label	(a)	0.6/	0/	0/	0.1/	0/	0.2/	0/	0.1/
		0.57/	0.071/	0/	0.14/	0/	0.14/	0/	0.071/
		0.7	0	0.085	0	0	0.085	0.13	0
	(b)	0.17/	0.33/	0.083/	0/	0.17/	0/	0.25/	0/
		0.077/	0.38/	0/	0/	0.077/	0.15/	0.23/	0.077/
		0.071	0.5	0	0	0.071	0.21	0.071	0.071
	(c)	0.067/	0/	0.33/	0/	0.27/	0.067/	0/	0.27/
		0.077/	0/	0.077/	0.46/	0.15/	0/	0.077/	0.15/
		0.18	0	0.46	0	0.36	0	0	0
	(d)	0.23/	0.23/	0.077/	0.38/	0/	0.077/	0/	0/
		0/	0/	0.23/	0.69/	0/	0/	0/	0.08/
		0	0	0.1	0.9	0	0	0	0
	(e)	0/	0/	0.4/	0/	0.33/	0/	0/	0.27/
		0/	0/	0/	0.55/	0.091/	0/	0.18/	0.18/
		0	0	0.3	0	0.4	0.2	0	0.1
	(f)	0.067/	0.13/	0.13/	0.2/	0/	0.2/	0.13/	0.13/
		0.28/	0.11/	0/	0.06/	0.17/	0.28/	0.11/	0/
		0.14	0.06	0	0	0	0.6	0.2	0
	(g)	0.11/	0.11/	0/	0/	0.11/	0.033/	0.22/	0.11/
		0.12/	0.12/	0/	0/	0.12/	0.12/	0.5/	0/
		0.077	0	0.15	0.077	0.15	0.077	0.38	0.077
	(h)	0.17/	0/	0.083/	0/	0.083/	0/	0.083/	0.58/
		0/	0/	0/	0/	0/	0/	0/	1/
		0	0	0	0	0	0	0	1

Each cell represents the results for ceiling/wall/dual radars.

**Table 6:** Selected feature parameters for the RF and SVM methods.

Radar	Selected Parameters
Ceiling radar	*v*_c-u-std_, *a*_c-u-max,_*a*_c-u-std,_*a*_c-m-max,_*j*_c-u-mean,_*j*_c-u-mean,_*j*_c-u-std,_*j*_c-m-max_
Wall radar	*v*_w-m-max_, *a*_w-u-min,_*a*_w-u-mean,_*a*_w-u-std,_*a*_w-m-max,_*j*_w-m-max,_*j*_w-m-min,_*j*_w-l-max_
Dual radar	*v*_w-u-min_, *v*_w-u-mean_*, v*_w-u-std,_*a*_c-u-max,_*a*_w-u-min,_*a*_w-u-mean,_*a*_w-u-std,_*a*_w-m-max,_*a*_w-m-min,_*a*_w-m-mean,_*a*_w-l-min,_*j*_c-u-std,_*j*_w-m-max,_*j*_w-l-max,_*j*_c-l-std_

*v*, *a*, and *j* denote velocity, acceleration, and jerk, respectively. The subscript “X-Y-Z” indicates radar type–envelope operation, X can be ceiling (c) or wall (w) radars. Y can be u, l, or m, indicating upper, lower, or power-weighted mean velocity, respectively; the parameter was extracted from *v*_u_(*t*), *v*_m_(*t*), and *v*_l_(*t*). Z indicates the calculation for the envelopes (std is standard deviation).

**Table 7:** Comparison of studies on restroom monitoring.

Study	Sensor	Problem	No. of Participants	Performance
[6]	Camera	Detection of dangerous situation	10	N. A. (Secure detection of dangerous situation continues for 60 s)
[9]	Thermal Sensor	Classification of normal/fall data	8	Accuracy over 95% (2-class classification)
[10]	Thermal Sensor	Classification of normal use/fall patterns	10	Accuracy: 97.8% (2-class classification)
[14]	Radar	Detection of dangerous state (such as falls)	3	Detection rate: 95%
[15]	Radar	Classification of normal/abnormal behaviors (including falls)	10	- Accuracy: 62.5% (7-class classification) - Fall classification rate: 83.3%
Our previous study [16]	Radar	Classification of eight behaviors (including falls)	21	- Accuracy: 83.2% (8-class classification) - Fall classification rate: 92.0%
This study	Radar	Classification of eight behaviors (including falls)	21	- Accuracy: 95.6% (8-class classification) - Fall classification rate: 100%

## Data Availability

The data presented in this study are available on request from the corresponding author. The data are not publicly available due to privacy issues.
